# Pulmonary metastatic colonisation and granulomas in NOX2‐deficient mice

**DOI:** 10.1002/path.5140

**Published:** 2018-09-19

**Authors:** Louise van der Weyden, Anneliese O Speak, Agnieszka Swiatkowska, Simon Clare, Andrea Schejtman, Giorgia Santilli, Mark J Arends, David J Adams

**Affiliations:** ^1^ Wellcome Sanger Institute, Wellcome Genome Campus Hinxton, Cambridge UK; ^2^ Molecular and Cellular Immunology (III), UCL Great Ormond Street Institute of Child Health London UK; ^3^ University of Edinburgh, Division of Pathology, Centre for Comparative Pathology, Cancer Research UK Edinburgh Centre, Institute of Genetics & Molecular Medicine, Western General Hospital Edinburgh UK

**Keywords:** metastasis, colonisation, reactive oxygen species, NOX2, granuloma, p22phox

## Abstract

Metastasis is the leading cause of death in cancer patients, and successful colonisation of a secondary organ by circulating tumour cells (CTCs) is the rate‐limiting step of this process. We used tail‐vein injection of B16‐F10 melanoma cells into mice to mimic the presence of CTCs and to allow for the assessment of host (microenvironmental) factors that regulate pulmonary metastatic colonisation. We found that mice deficient for the individual subunits of the NADPH oxidase of myeloid cells, NOX2 (encoded by *Cyba*, *Cybb*, *Ncf1*, *Ncf2*, and *Ncf4*), all showed decreased pulmonary metastatic colonisation. To understand the role of NOX2 in controlling tumour cell survival in the pulmonary microenvironment, we focused on *Cyba*‐deficient (*Cyba* ^*tm1a*^) mice, which showed the most significant decrease in metastatic colonisation. Interestingly, histological assessment of pulmonary metastatic colonisation was not possible in *Cyba* ^*tm1a*^ mice, owing to the presence of large granulomas composed of galectin‐3 (Mac‐2)‐positive macrophages and eosinophilic deposits; granulomas of variable penetrance and severity were also found in *Cyba* ^*tm1a*^ mice that were not injected with melanoma cells, and these contributed to their decreased survival. The decreased pulmonary metastatic colonisation of *Cyba* ^*tm1a*^ mice was not due to any overt defects in vascular permeability, and bone marrow chimaeras confirmed a role for the haematological system in the reduced metastatic colonisation phenotype. Examination of the lymphocyte populations, which are known key regulators of metastatic colonisation, revealed an enhanced proportion of activated T and natural killer cells in the lungs of *Cyba* ^*tm1a*^ mice, relative to controls. The reduced metastatic colonisation, presence of granulomas and altered immune cell populations observed in *Cyba* ^*tm1a*^ lungs were mirrored in *Ncf2*‐deficient (*Ncf2* ^*tm1a*^) mice. Thus, we show that NOX2 deficiency results in both granulomas and the accumulation of antitumoural immune cells in the lungs that probably mediate the decreased pulmonary metastatic colonisation. © 2018 The Authors. *The Journal of Pathology* published by John Wiley & Sons Ltd on behalf of Pathological Society of Great Britain and Ireland.

## Introduction

Metastasis is the leading cause of death in cancer patients, and is a multistep process that involves invasion of the basement membrane by the tumour cells, intravasation, survival in the circulation, extravasation, and proliferation at a secondary site 1. The successful colonisation of a secondary organ by circulating tumour cells (CTCs) is the rate‐limiting step in this process 2. The lung is a common site of metastasis for many tumour types, and is thus a clinically relevant organ for investigation of colonisation factors. Large‐scale sequencing has identified genes that are differentially expressed in metastatic cells relative to the primary tumour, and many studies have examined the role of tumour cell intrinsic factors that can regulate metastasis; however, less work has been performed to investigate tumour cell extrinsic factors, i.e., the role of the host, which includes microenvironmental factors such the stroma, endothelial cells, and the immune system.

Tail‐vein injection of tumour cells into mice (providing CTCs in the blood) allows for assessment of extrinsic factors that regulate pulmonary metastatic colonisation 3. As previously published, we used this method to robustly screen 810 genetically modified mouse lines to identify 23 novel microenvironmental (‘tumour cell extrinsic’) regulators of pulmonary metastatic colonisation 4, 5, with genes whose loss of function results in decreased metastatic colonisation representing potential drug targets. In this screen, we identified *Cyba*‐deficient, *Cybb*‐deficient and *Ncf2*‐deficient mice as showing reduced metastatic colonisation. These genes encode protein subunits of the NADPH oxidase NOX2.

Members of the NADPH oxidase family of proteins, i.e. NOX1–NOX5, DUOX1, and DUOX2, are important enzymatic sources of reactive oxygen species (ROS). The seven family members differ in their subunit composition, type of ROS release, mode of activation, and tissue expression 6. NOX2, known as the phagocyte NADPH oxidase (‘phox’), consists of two membrane‐spanning subunits, i.e. gp91phox and p22phox (encoded by *CYBB* and *CYBA*, respectively), and three cytoplasmic subunits, i.e. p47phox, p67phox, and p40phox (encoded by *NCF1*, *NCF2*, and *NCF4,* respectively); upon stimulation, the cytoplasmic subunits recruit a GTPase (either Rac1 or Rac2), bind the transmembrane components, and activate the enzymatic complex to transport electrons from NADPH to oxygen, thus generating ROS, which play a role in phagocytic host defence against invading organisms 7, 8.

Maintaining the balance between the generation and elimination of ROS is critical; both continuously elevated and insufficient ROS levels can be detrimental to health 9. A defect in any of the five genes encoding NOX2 subunits results in chronic granulomatous disease (CGD) – a rare inherited immunodeficiency syndrome characterised by recurrent and life‐threatening infections with bacterial and fungal pathogens, often with granuloma formation 10, and the most common site of involvement being the lungs 11. Whereas ROS are critical for microbial killing in the phagosome, phagocytes also produce large amounts of extracellular ROS, which can have immunosuppressive effects, such as reducing T‐cell immune responses 12 and natural killer (NK) cell function 13. In addition, phagocytes are not the only cell types expressing NOX2; dendritic cells express NOX2 on antigen‐containing endosomes and phagosomes (critical for the process of antigen cross‐presentation and the initiation of cytotoxic T‐cell immune responses) 14, 15. Thus, the role of NOX2 in controlling tumour cell survival in the pulmonary microenvironment is complex.

In order to understand the role of NOX2 in regulating the ability of CTCs to effectively colonise the lung, as well as gain mechanistic insights, we utilised mutant mice deficient for each one of the five subunits of the NOX2 complex to examine their ability to regulate pulmonary metastatic colonisation and assess the immune composition of their pulmonary microenvironment.

## Materials and methods

### Mice

*Cyba* [*Cyba* ^*tm1a(EUCOMM)Wtsi*^ (ES cell clone: EPD0372_5_B08); hereafter referred to as *Cyba* ^*tm1a*^] and *Ncf2* [*Ncf2* ^*tm1a(EUCOMM)Wtsi*^ (ES cell clone: EPD0240_5_B03); hereafter referred to as *Ncf2* ^*tm1a*^] mutant alleles were generated by EUCOMM and transmitted through the germline at the Wellcome Sanger Institute (WSI). Mice were genotyped by polymerase chain reaction (PCR) with either a generic strategy to detect the Neo cassette or an allele‐specific short‐range PCR strategy 16. The generation, genotyping and characterisation of *Cybb* (*Cybb* ^*tm1Din*/*J*^) 17, *Ncf1* (*Ncf1* ^*m1J*/*J*^) 18 and *Ncf4* (*p40phox*) 19 mutant mice have been previously described. Phenotyping of all mutant mice was performed on homozygotes. The care and use of all mice in this study were in accordance with the UK Animals in Science Regulation Unit's Code of Practice for the Housing and Care of Animals Bred, Supplied or Used for Scientific Purposes, and the Animals (Scientific Procedures) Act 1986 Amendment Regulations 2012, and all procedures were performed under a UK Home Office Project Licence, which was reviewed and approved by the Sanger Institute's Animal Welfare and Ethical Review Body. Housing and husbandry conditions of the mice and general experimental design (allocation of mice to a treatment group, blinding, statistical power, etc.) were as detailed previously 4.

### Cell lines

The mouse melanoma B16‐F10 cell line was purchased from the American Type Culture Collection (CRL‐6475), and the metastatic mouse mammary cancer EO771.LMB cell line 20 was a gift from R. L. Anderson (Peter MacCallum Cancer Centre, Australia). The generation of the B16‐F10–mCherry cell line has been described previously 4. All cells were maintained in Dulbecco's modified Eagle's medium with 10% (v/v) fetal bovine serum and 2 mm glutamine, and 100 U/ml penicillin/streptomycin (with the addition of 20 mm HEPES for EO771.LMB cells), at 37 °C in 5% CO_2_ (all tissue culture reagents were from Gibco, Waltham, MA, USA). All cell lines were screened for the presence of mycoplasma and mouse pathogens (at Charles River Laboratories, Wilmington, MA, USA) before being cultured, and were never cultured for more than five passages. None of the cell lines used appear in the International Cell Line Authentication Committee database.

### Experimental metastasis assay

B16‐F10 (4 × 10^5^) and EO771.LMB (4 × 10^5^) cells resuspended in 0.1 ml of phosphate‐buffered saline (PBS) were injected into the tail veins of 6–12‐week‐old sex‐matched syngeneic control and mutant mice. After 10 days, the mice were killed and their lungs were removed, and the metastatic tumour burden was determined either by macroscopic counting (for B16‐F10 cells) or mCherry quantitative PCR (qPCR) (for EO771.LMB cells) (as described previously) 3.

### Histology and immunohistochemistry

Mouse tissues were rinsed with PBS and then placed in 10% neutral‐buffered formalin (NBF) for a minimum of 24 h before being dehydrated and embedded in paraffin; sections were then cut and stained with haematoxylin and eosin (H&E), according to standard histology protocols. Immunohistochemistry was performed after standard antigen retrieval (20 min at 100 °C in citrate buffer, pH 6) and blocking (peroxidase‐blocking solution; Dako, Santa Clara, CA, USA) procedures. The primary antibodies used were: rat anti‐mouse galectin‐3 (Mac‐2) antibody (1:1000 dilution; clone M3/38; Cedarlane, Burlington, Ontario, Canada) and rabbit anti‐S100β antibody (1:250 dilution; clone EP1576Y; Abcam, Cambridge, UK). The secondary antibodies used were: biotinylated goat anti‐rat IgG mouse absorbed antibody and biotinylated goat anti‐rabbit IgG antibody (BA‐4000 and BA‐1000 respectively; both 1:200 dilution; Vector Laboratories, Peterborough, UK), with visualisation being achieved with 3,3′‐diaminobenzine and counterstaining with haematoxylin.

### Bone marrow chimaeras

Wild‐type (F_1_ CD45.1/2 heterozygous congenic) mice were subjected to 2 × 4.2‐Gy whole‐body gamma irradiation followed by tail vein administration of 4 × 10^6^ bone marrow cells from either wild‐type (CD45.1 congenic) or mutant (*Cyba* ^*tm1a*^ or *Ncf2* ^*tm1a*^; CD45.2) mice. Six weeks after transplantation, a tail‐vein blood sample was taken to assess the relative proportions of CD45.1 and CD45.2 cells (to assess effective reconstitution of the donor bone marrow), and 5 days later an experimental metastasis assay was performed as described above.

### Preparation of tissue cell suspensions and flow cytometry

Mice were given a terminal dose of pentobarbitone intraperitoneally before being perfused with 10 ml of PBS by cardiac puncture, and the lungs and spleen were dissected out. To determine the number of melanoma cells present in the lungs, the mice were dosed with 1 × 10^6^ B16‐F10–mCherry cells 90 min before perfusion. Preparation of a single‐cell suspension for each tissue, enrichment of leukocytes within the cell suspension and staining of the leukocytes with multicolour antibody cocktails and viability dyes was performed as described previously 4. All samples were analysed on an LSRFortessa SORP instrument (BD Biosciences, Franklin Lakes, NJ, USA) that was calibrated with BD Cytometer Setup and Tracking Beads and software. Compensation was determined with Ultracomp eBeads (eBioscience, San Diego, CA, USA) for all antibodies, and ArC amide‐binding beads (Invitrogen, Carlsbad, CA, USA) for live/dead stains. Data acquisition was controlled with BD FACSDiva version 8.0.1 software (BD Biosciences). Data from doublets (determined with FSC‐A versus FSC‐H gates, and SSC‐H versus SSC‐W gates), dead cells (determined with the viability dye) and debris (determined with FSC‐A versus SSC‐A gates) were excluded. A leukocyte gate was set with CD45 and SSC‐A, and all cell subsets were reported as the percentage of this parent gate. All samples were analysed with FlowJo X (FlowJo LLC, Ashland, OR, USA).

### Statistical analysis

Data were analysed with GraphPad Prism 6 software (version 6.04) (GraphPad Software, San Diego, CA, USA), and are shown as dot plots with standard error of the mean error bars (or standard deviation error bars for the experimental metastasis assay, for both 90‐min and 10‐day results). Groups were compared by the use of unpaired, two‐tailed Student's *t*‐tests or Mann–Whitney *U*‐tests as appropriate, or two‐way anova with Bonferroni's multiple comparisons correction, as detailed in the figure legends. Survival was calculated with the log‐rank (Mantel–Cox) test. Differences between groups were considered to be significant at *p* ≤ 0.05.

## Results

### NADPH oxidase‐deficient mice show decreased pulmonary metastatic colonisation

**Figure 1:**
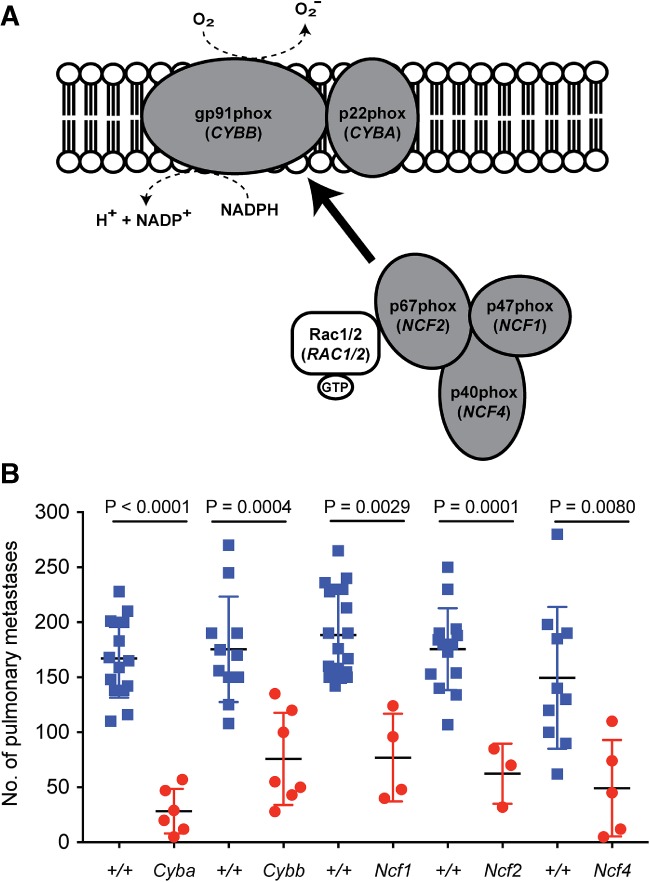
The NADPH oxidase NOX2 complex. (A) Schematic representation of the subunits of NOX2. NOX2 requires association with cytosolic regulatory subunits for its activation, as indicated. (B) Experimental pulmonary metastasis numbers in mice deficient for individual components of the NOX2 complex. Female wild‐type (blue squares) and homozygous mutant (red circles) mice aged 6–10 weeks were tail‐vein‐dosed with B16‐F10 melanoma cells, and the number of pulmonary metastases was counted 10 days later. Symbols represent individual mice from a single cohort, and statistical evaluation was performed with a Mann–Whitney *U*‐test. Data are representative of at least three independent cohorts.

The NOX2 complex is composed of two membrane‐bound subunits (encoded by *Cyba* and *Cybb*) and three cytosolic subunits (encoded by *Ncf1*, *Ncf2*, and *Ncf4*; Figure 1A). Mice deficient (homozygotes) in any of the NOX2 subunits showed a reduced number of metastatic foci in the lungs, underscoring the importance of NOX2 in regulating pulmonary metastatic colonisation (Figure 1B). As the most reduced level of colonisation was seen in *Cyba*‐deficient mice, we decided to focus on the role of Cyba in the pulmonary microenvironment.

### *Cyba*‐deficient mice develop pulmonary granulomas

**Figure 2:**
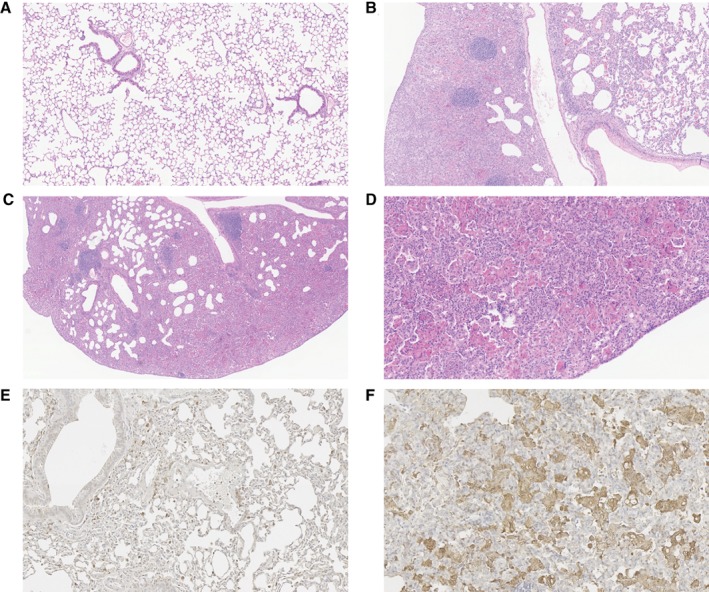
Granulomas in *Cyba*‐deficient mice. (A) Representative image of a lung from a wild‐type mouse showing no granulomas or eosinophilic deposits (magnification: ×100). (B) Representative image of a *Cyba* ^*tm1a*^ lung showing a moderate granuloma (magnification: ×100). (C) Representative image of a *Cyba* ^*tm1a*^ lung showing extensive severe granuloma formation with scattered lymphoid aggregates (magnification: ×50). (D) Representative image of a *Cyba* ^*tm1a*^ lung showing a severe granuloma with the presence of eosinophilic crystalline deposits (magnification: ×200). (E and F) Representative images of a wild‐type lung (E) and a *Cyba* ^*tm1a*^ lung (F) showing positive immunohistochemical staining for Mac‐2 (magnification: ×200).

To confirm the macroscopic counts of lungs from B16‐F10‐dosed mice, lung colonisation was assessed by examination of H&E‐stained sections of all five lobes of the lung. However, it was not possible to determine the metastatic burden in the lungs of *Cyba* ^*tm1a*^ mice, owing to the presence of very large confluent granulomas, composed of numerous macrophages, including activated and hyperactivated macrophages/histiocytes, as well as some evidence of eosinophilic crystalline debris, in the interstitial stroma of the lung parenchyma (Figure 2A–D). Thus, we used S100 immunohistochemistry to identify the presence of the B16‐F10 melanoma cells, and found no evidence of single melanoma cells or small clusters of melanoma cells within the granulomas (supplementary material, Figure S1). To assess whether these granulomas were induced by the presence of the tumour cells in the lung, histopathological analysis of the lungs was performed on age‐matched mice that had not been injected with tumour cells. In contrast to wild‐type mice, *Cyba* ^*tm1a*^ mice showed the presence of granulomas and eosinophilic crystals, with variable degrees of severity and penetrance (Table 1; Figure 2A–D). The granulomas were composed of macrophages, as confirmed by anti‐Mac‐2 immunohistochemistry (Figure 2E,F).

**Table 1:** Granuloma formation and eosinophilic crystal deposits in the lungs of 6–9‐week‐old wild‐type and *Cyba* ^*tm1a*^ mice

	**Granulomas**				**Eosinophilic crystals**	
**Genotype**	**Absent**	**Mild**	**Moderate**	**Severe**	**Absent**	**Present**
Wild‐type	8/8	–	–	–	8/8	–
*Cyba* ^*tm1a*^	4/8	2/8	1/8	1/8	4/8	4/8

Lungs from wild‐type and *Cyba* ^*tm1a*^ mice at 6–9 weeks of age were collected into 10% NBF and then histologically processed to allow assessment of an H&E‐stained slide containing a longitudinal section of all five lobes. The lungs were graded as mild, moderate or severe on the basis of the strongest phenotype seen in at least one of the five lobes. The grading of the severity of the eosinophilic granules present ‘matched’ that of the granulomas.

**Figure 3:**
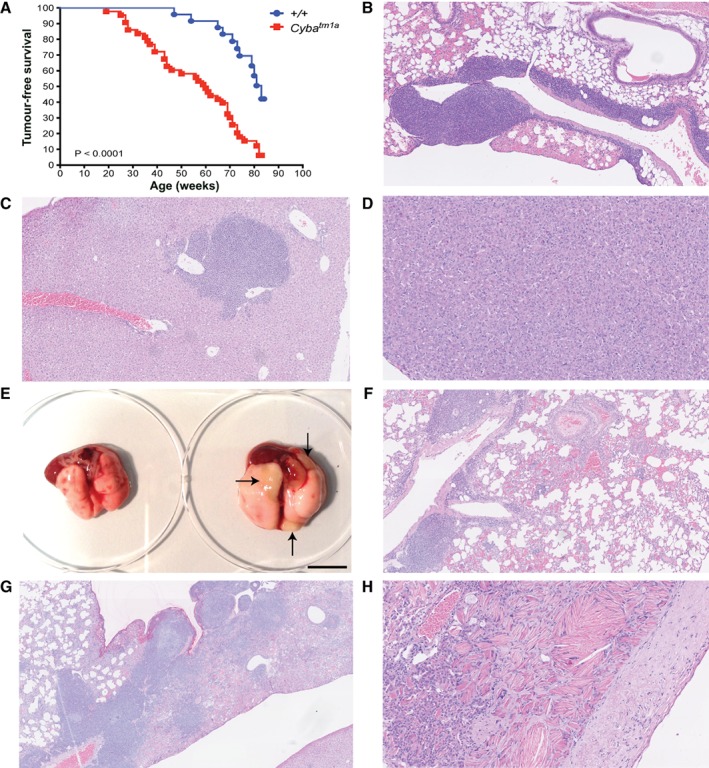
Aged *Cyba*‐deficient mice. (A) Kaplan–Meier curve showing tumour‐free survival of wild‐type (blue circles) and *Cyba*‐deficient (red squares) mice. Statistical analysis was performed with a log‐rank (Mantel–Cox) test. (B) Representative image of a lymphoma in the lung of a *Cyba* ^*tm1a*^ mouse (magnification: ×100). (C) Representative image of a lymphoma in the liver of a *Cyba* ^*tm1a*^ mouse (magnification: ×100). (D) Representative image of a hepatocellular carcinoma in a *Cyba* ^*tm1a*^ mouse (magnification: ×200). (E) Representative macroscopic image of the lungs from a wild‐type mouse (left) and a *Cyba* ^*tm1a*^ mouse (right) at the time of necropsy. Arrows show the macroscopically visible granulomas present in the *Cyba* ^*tm1a*^ lung. Scale bar: 1 cm. (F) Representative image of a *Cyba* ^*tm1a*^ lung showing a moderate granuloma (magnification: ×100). (G) Representative image of a *Cyba* ^*tm1a*^ lung showing a severe granuloma with eosinophilic crystalline deposits. Note the coexisting lymphoma (magnification: ×40). (H) Representative image of a *Cyba* ^*tm1a*^ lung showing eosinophilic crystals and multi‐crystal deposits with associated pleural plaque formation (magnification: ×200).

Concomitant with this, *Cyba*‐deficient mice showed significantly decreased survival as compared with wild‐type mice (median survival of 60 weeks and 83 weeks, respectively), due to a combination of increased tumour incidence (Figure 3A) and widespread pulmonary granulomas inducing dyspnoea or respiratory failure. The tumours were primarily lymphomas (Table 2), both in the lungs and in other tissues that were examined (typically the thymus, spleen, liver, kidney, and lymph nodes; Figure 3B,C), with some cases of hepatocellular carcinoma being seen (Figure 3D). Of the 46 *Cyba* ^*tm1a*^ mice that were killed on humane grounds, 38 were killed (82%) because of irregular or laboured breathing, and their lungs showed the presence of granulomas and eosinophilic crystals; three of 46 (6.5%) were graded as mild, and 43 of 46 (93.5%) were graded as moderate–severe (Figure 3E–H). Interestingly, six of 46 (13%) mice did not show any evidence of a tumour (in the lungs or other tissues) at the time of necropsy, but granulomas and eosinophilic crystals were present. Granulomas and eosinophilic crystals were never seen in the lungs of aged wild‐type mice.

**Table 2:** Diagnosis of wild‐type and *Cyba* ^*tm1a*^ ‘tumour watch’ mice

Genotype	Lymphoma	Hepatocellular carcinoma	Harderian gland hyperplasia	No tumour	Pulmonary granulomas	Pulmonary eosinophilic crystals
Wild‐type	13/14	1/14	1/14	0/14	0/14	0/14
*Cyba* ^*tm1a*^	39/46	1/46	2/46*	6/46	46/46	46/46

After 85 weeks, 14 of 30 wild‐type mice and 46 of 50 homozygous *Cyba* ^*tm1a*^ mice had been killed on humane grounds and necropsies had been performed for histopathological diagnosis. *One mouse also had a Harderian gland adenoma.

### *Cyba*‐deficient mice show decreased pulmonary metastatic colonisation

**Figure 4:**
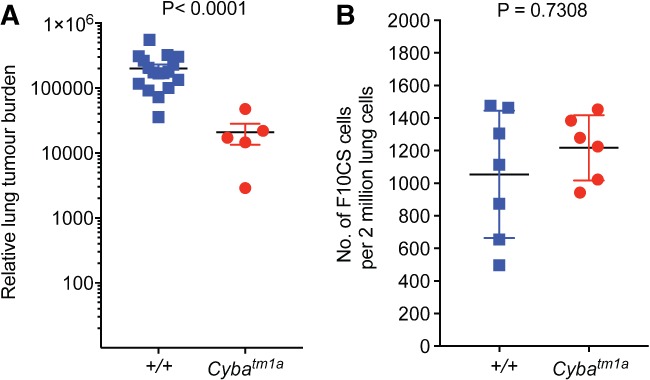
*Cyba*‐deficient mice show decreased pulmonary metastatic colonisation. (A) Experimental metastasis assay using EO771.LMB breast cancer cells administered to wild‐type (*+*/*+*) and *Cyba* ^*tm1a*^ mice, in which pulmonary metastatic colonisation was measured by mCherry qPCR and represented as relative lung tumour burden per mouse. (B) Number of F10CS melanoma cells present in the lungs of wild‐type (*+*/*+*) and *Cyba* ^*tm1a*^ mice, 90 min after tail‐vein administration. Symbols represent individual mice, and statistical analysis was performed with a Mann–Whitney *U*‐test. Data are representative of at least three independent cohorts.

In order to be able to confirm that the reduced pulmonary metastatic colonisation phenotype we observed was not specific to the B16‐F10 melanoma cell line, and to circumvent the need for histological assessment of tumour burden, we used the EO771.LMB breast cancer cell line expressing mCherry 20. Pulmonary metastatic colonisation in mice that had been given EO771.LMB cells via the tail‐vein was assessed by qPCR on DNA extracted from the lungs of the mice [determined by the level of mCherry DNA (present only in the tumour cells) relative to that of vimentin DNA (present in all cells)]. As shown in Figure 4A, relative to wild‐type controls, *Cyba* ^*tm1a*^ mice showed reduced tumour burden in their lungs 10 days after being tail‐vein‐dosed with EO771.LMB cells.

Given that ROS can participate in vascular cell function and that *Cyba* is also expressed in endothelial cells 21, we decided to assess whether the reduced metastatic phenotype was due to an inability of the tumour cells to extravasate from the bloodstream into the lung. As shown in Figure 4B, 90 min after tail‐vein administration of F10CS cells (B16‐F10 cells expressing mCherry), both wild‐type and *Cyba* ^*tm1a*^ mice had similar numbers of F10CS cells present in their lungs, indicating that the reduced pulmonary metastatic colonisation observed in *Cyba* ^*tm1a*^ mice was not due to differences in extravasation from the pulmonary capillaries.

### Role of the immune composition of the lungs

**Figure 5:**
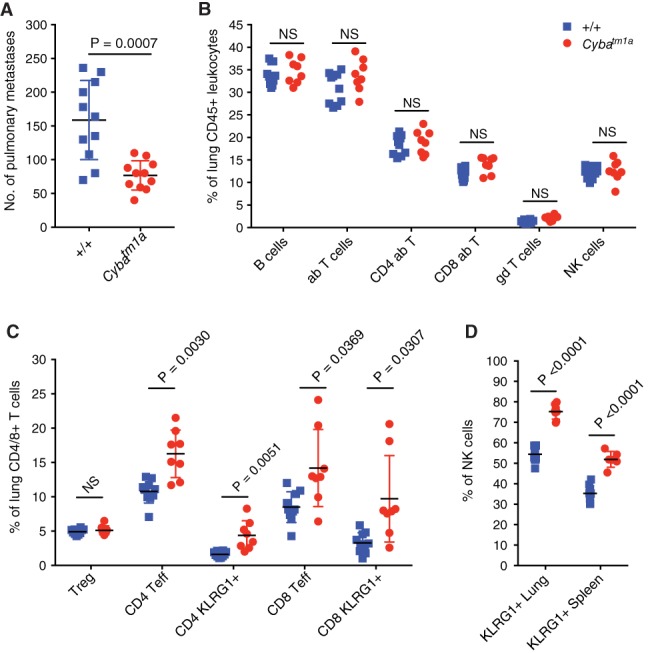
The role of the immune system in *Cyba*‐deficient mice. (A) Experimental metastasis assay in irradiated wild‐type mice repopulated with wild‐type (*+*/*+*) or *Cyba* ^*tm1a*^ bone marrow and tail‐vein‐dosed with B16‐F10 melanoma cells. (B and C) The percentages of lymphocyte subsets in the lungs of +/+ and *Cyba* ^*tm1a*^ mice. Data are shown as percentage of alive CD45^+^ lung cells present (B) or percentage of parent CD4^+^ and CD8^+^ T cells (C). (D) The percentage of KLRG1^+^ NK cells in the lungs and spleen of +/+ (blue squares) and *Cyba* ^*tm1a*^ (red circles) mice. Symbols represent individual mice; the horizontal bar indicates the mean. *P* values were obtained with Mann–Whitney *U*‐tests, and the data are representative of two independent experiments (A), or *P* values were obtained with two‐tailed unpaired *t*‐tests adjusted by use of the Holm–Šídák method with *α* set to 5%, and the data are representative of two independent experiments (B–D). ab, alpha beta T cell; gd, gamma delta T cell; NS, not significant; Teff, effector T cell; Treg, regulatory T cell.

To determine whether components of the immune system were responsible for the reduced pulmonary metastatic colonisation in *Cyba* ^*tm1a*^ mice, we lethally gamma‐irradiated wild‐type mice (to ablate their bone marrow stem cells) and tail‐vein‐administered bone marrow isolated from either wild‐type or *Cyba* ^*tm1a*^ mice. After 6 weeks to allow reconstitution of the donor bone marrow, the irradiated recipients were used in an experimental metastasis assay with B16‐F10 cells to determine whether this could recapitulate the phenotype seen in *Cyba* ^*tm1a*^ mice. Mice that received *Cyba* ^*tm1a*^ donor bone marrow showed reduced pulmonary metastatic colonisation relative to those that received bone marrow from wild‐type mice (Figure 5A). This confirmed that it was a component of the haematopoietic system that was responsible, as least in part, for mediating the reduced pulmonary metastatic colonisation. Interestingly, the lungs of mice that received *Cyba* ^*tm1a*^ donor bone marrow did not show the presence of any granulomas or eosinophilic crystalline deposits (*n* = 0/8 mice), suggesting that this phenotype is initiated and/or partly mediated by a non‐haematopoietic component in addition to macrophage involvement.

Given that the haematopoietic system was implicated in mediating metastatic colonisation, and that lymphocytes are known to kill tumour cells, we collected the lungs of mice that had been perfused with saline to remove blood from the blood vessels, and performed immunophenotyping of the lymphocyte populations present. As shown in Figure 5B, *Cyba* ^*tm1a*^ and wild‐type mice showed no differences in the relative percentages of B lymphocytes, T lymphocytes or NK cells in their lungs. However, regarding the T cells present in the lungs, *Cyba* ^*tm1a*^ mice showed a significantly higher proportion than wild‐type controls of antitumoural effector memory T cells (CD44^hi^CD62L^lo^) that were activated/antigen‐experienced (KLRG1+; Figure 5C). Interestingly, this phenotype was not observed in the spleen (data not shown). Similarly, regarding the NK cells present in the lungs, those from *Cyba* ^*tm1a*^ mice were more activated than those from wild‐type mice, and this was also observed in the spleen (Figure 5D).

### *Cyba*‐deficient mouse pulmonary phenotypes were mirrored in *Ncf2*‐deficient mice

**Figure 6:**
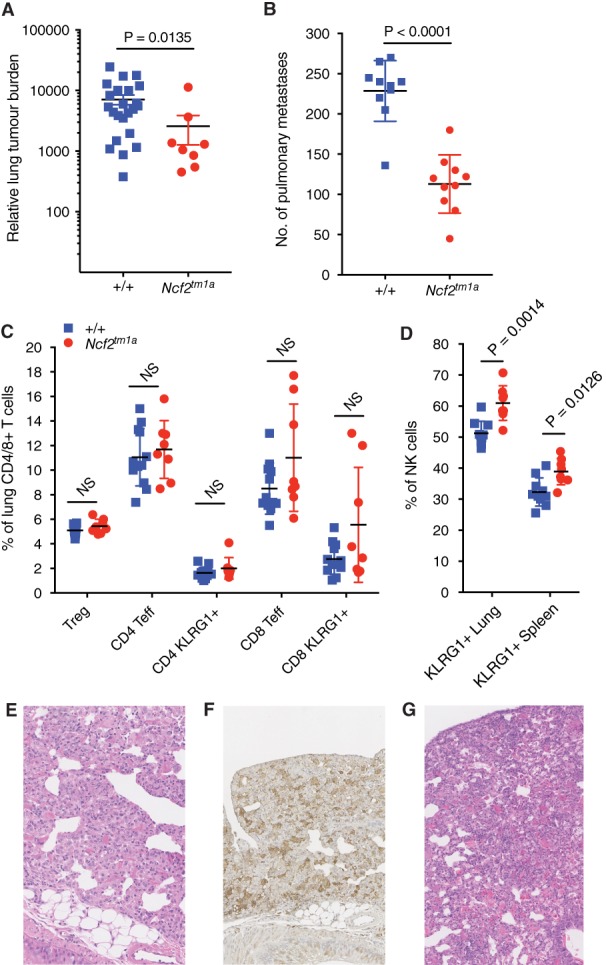
The pulmonary metastatic colonisation, lymphocyte populations and granulomas in *Ncf2*‐deficient mice. (A) Experimental metastasis assay using EO771.LMB breast cancer cells administered to wild‐type (*+*/*+*) and *Ncf2* ^*tm1a*^ mice, in which pulmonary metastatic colonisation was measured by mCherry qPCR and represented as relative lung tumour burden per mouse. (B) Experimental metastasis assay in irradiated wild‐type mice repopulated with wild‐type (*+*/*+*) or *Ncf2* ^*tm1a*^ bone marrow, and tail‐vein‐dosed with B16‐F10 melanoma cells. (C) The percentage of lymphocyte subsets in the lungs of +/+ and *Ncf2* ^*tm1a*^ mice, shown, as percentage of alive CD45^+^ lung cells present. (D) The percentage of KLRG1^+^ NK cells in the lungs and spleen of +/+ (blue squares) and *Ncf2* ^*tm1a*^ (red circles) mice. (E) Representative image of an *Ncf2* ^*tm1a*^ lung showing a mild granuloma and eosinophilic crystals (magnification: ×200). (F) The same *Ncf2* ^*tm1a*^ lung showing positive immunohistochemical staining for Mac‐2 (magnification: ×200). (G) Representative image of an *Ncf2* ^*tm1a*^ lung showing severe granuloma formation and eosinophilic crystals (magnification: ×200). Symbols represent individual mice; the horizontal bar indicates the mean. *P* values were obtained with Mann–Whitney *U*‐tests, and the data are representative of two independent experiments (A and B), or *P* values were obtained with two‐tailed unpaired *t*‐tests adjusted by use of the Holm–Šídák method with *α* set to 5%, and the data are representative of two independent experiments (C and D). NS, not significant; Teff, effector T cell; Treg, regulatory T cell.

As the p22phox (*Cyba*) subunit of NOX2 is present in other NOX complexes, specifically NOX1, NOX3, and NOX4, we next investigated whether the pulmonary phenotypes that we observed in *Cyba*‐deficient mice were also present in *Ncf2*‐deficient mice, as the p67phox (*Ncf2*) subunit is unique to NOX2. *Ncf2*‐deficient mice showed decreased pulmonary metastatic colonisation by EO771.LMB breast cancer cells relative to wild‐type mice (Figure 6A). Similarly, lethally irradiated wild‐type mice that received *Ncf2* ^*tm1a*^ donor bone marrow showed decreased pulmonary metastatic colonisation by B16‐F10 melanoma cells relative to those that received donor bone marrow from wild‐type mice (Figure 6B). Immunophenotyping of the lungs showed that *Ncf2*‐deficient mice had an increased percentage of activated effector memory T cells relative to wild‐type mice (Figure 6C), although the difference was not statistically significant and not as pronounced as the difference seen with *Cyba*‐deficient mice (Figure 5). Similarly, regarding the NK cells present in the lungs, those from *Ncf2* ^*tm1a*^ mice were more activated than those from wild‐type mice, and this phenotype was also observed in the spleen (Figure 6D). Finally, granulomas and eosinophilic crystal deposits (of mild grade) were also observed in the lungs of four of eight (50%) 6–9‐week‐old *Ncf2* ^*tm1a*^ mice (Figure 6E,F). We were unable to perform a complete ageing study on *Ncf2* ^*tm1a*^ mice, as many of the cohort had to be humanely killed at an early age (31 ±6 weeks) because of excessive scratching of their back skin, generating open wounds (17/31, 55%). Histological analysis of tissues collected at the time of necropsy showed no evidence of malignancy in the majority of cases (only one case of lymphoma); however, the presence of granulomas and crystals in their lungs was noted in 88% (15/17) of cases, ranging from mild to severe. Of the remaining mice in the ageing study, 10 of 14 (71%) had to be killed because of ulceration of the eye at an early age (six of these mice were also excessively scratching; 29 ±13 weeks; an enlarged Harderian gland makes the eye protrude, thus making it difficult to close the eyelid), and Harderian gland hyperplasia was confirmed histologically in all cases [with one case having a concomitant lymphoma, and 50% (5/10) of the cases showing moderate–severe granulomas and crystals]. There were only four mice in the ageing study that were killed at the time when they showed clinical signs of being unwell (82 ±6 weeks), and only one of these was killed because of dyspnoea [histological analysis confirmed the presence of severe granulomas and eosinophilic deposits (Figure 6G); the other three mice had lymphoma, with one mouse having a concomitant liver angiosarcoma, and all had moderate–severe granulomas and crystals].

## Discussion

Decreased pulmonary metastatic colonisation has been previously reported in *Cybb*‐deficient mice 2, 22, 23; however we show here that mice deficient for any of the five subunits of NOX2 (*Cybb*‐deficient, *Cyba‐*deficient, *Ncf1‐*deficient, *Ncf2‐*deficient and *Ncf4*‐deficient mice) have decreased pulmonary metastatic colonisation. To gain mechanistic insights into how loss of NOX2 could be affecting pulmonary colonisation by CTCs, we chose two of these mutant mouse lines for further investigation. We chose the *Cyba* ^*tm1a*^ and *Ncf2* ^*tm1a*^ lines because they: (1) were generated at the WSI (and so were on the same strain/genetic background); (2) represent a transmembrane component versus a cytosolic component of NOX2 (respectively); and (3) represent a component present in other NOX complexes versus one found only in NOX2 (respectively).

Decreased pulmonary metastatic colonisation was seen in irradiated wild‐type mice that received *Cyba* ^*tm1a*^ or *Ncf2* ^*tm1a*^ bone marrow (but not wild‐type bone marrow), suggesting that this phenotype is primarily mediated by the immune system. Immunophenotyping of the lungs of *Cyba* ^*tm1a*^ mice showed an increased percentage of activated T effector cells and NK cells. Interestingly, increased levels of activated T cells were seen only in the lungs of the mice, not in their spleens, suggesting that the lungs were providing a microenvironment conducive to the activation of the T cells, as opposed to a systemic increase in the percentage of activated T cells. In contrast, the increase in the percentage of activated NK cells was seen in both lung and spleen. Both T cells and NK cells are known to have strong antitumoural functions, and so could be responsible for mediating the reduced pulmonary metastatic colonisation seen in these mice. Given that *Ncf2* ^*tm1a*^ mice showed only a small increase in the percentage of activated T lymphocytes, but showed a significant increase in the percentage of activated NK cells, it is tempting to speculate that NK cells are the main mediators of this phenotype. Metastatic colonisation is strongly regulated by NK cells, and *in vivo* depletion of NK cells in wild‐type mice results in significantly increased pulmonary metastatic colonisation 3. Interestingly, *in vivo* depletion of NK cells in *Cybb*‐deficient mice was recently shown to result in increased pulmonary metastatic colonisation by B16‐F10 cells, with the degree of metastasis more than doubling in NK‐cell‐depleted *Cybb*‐deficient mice relative to NK‐cell‐depleted wild‐type mice, which the authors suggested implied increased functionality of NK cells in the absence of NOX2 23. *In vivo* depletion of NK cells in both *Cyba* ^*tm1a*^ and *Ncf2* ^*tm1a*^ mice gave inconsistent results, despite being able to demonstrate the effectiveness of the NK1.1 antibody treatment by confirming loss of NK cells in the blood (data not shown). This variability may be due to only a partially penetrant role for CD8+ T‐cells, or may be due to the granulomas. Granulomas are present in *Cyba* ^*tm1a*^ and *Ncf2* ^*tm1a*^ mice as early as 6–9 weeks of age with varying penetrance and severity, and these pools of macrophages could release signals that have an effect on the ability of the tumour cells to undergo metastatic colonisation and/or affect the immune cells present in the lung – thus adding a confounding factor to the analysis of *Cyba* ^*tm1a*^ and *Ncf2* ^*tm1a*^ mice that would not exist in wild‐type mice. We noted that the granulomas were frequently macroscopically obvious in NK cell‐depleted *Cyba* ^*tm1a*^ mice, and histological analysis confirmed that NK cell depletion resulted in both increased presence and increased size of granulomas in *Cyba* ^*tm1a*^ mice (data not shown), suggesting that the NK cell depletion enhanced granuloma formation; also, NK cell depletion has been reported to cause an increase in granuloma diameter in several mouse models of granulomatous inflammation, without any effect on T‐cell subsets or proliferative ability 24.

The stronger metastatic colonisation and protumoural immune cell population phenotype seen in *Cyba* ^*tm1a*^ mice relative to *Ncf2* ^*tm1a*^ mice may be due to the fact that the Cyba subunit is present in other NOX complexes, specifically NOX1, NOX3, and NOX4. For example, unlike *Ncf2*‐deficient mice, *Cyba*‐deficient mice have a balance disorder (see the IMPC website: http://www.mousephenotype.org/data/genes/MGI:1316658#section-associations and 25) caused by the aberrant development of the gravity‐sensing organs, as NOX3 is primarily expressed in the inner‐ear epithelium 25. *Cyba*‐deficient mice (*nmf33* mice) have been shown to develop a CGD‐like immune defect, as the inability of the phagocytes to produce bacteria‐destroying ROS means that these mice have increased susceptibility to bacterial infections, dying a few days after intratracheal inoculation of *Burkholderia cepacia*; their lung tissue was greatly damaged by necrotising pneumonia, and the alveolar spaces were filled with neutrophil granulocytes, macrophages, and cell debris, in contrast to only minimal granulocytic infiltration into the peribronchial and perivascular areas in lungs from wild‐type mice 25). In agreement with this, *Cyba* ^*tm1a*^ mice have been shown to die a few days after intravenous administration of *Salmonella enterica* serovar Typhimurium 26. However, this is the first report of the presence of macrophage infiltration in the lungs resulting in the formation of granulomas with eosinophilic crystalline deposits in *Cyba*‐deficient (and *Ncf2*‐deficient) mice that have not been deliberately exposed to a microorganism and that were housed in a specific pathogen‐free (SPF) environment. Histopathological analysis of the lungs from 6–9‐week‐old *Cyba* ^*tm1a*^ and *Ncf2* ^*tm1a*^ mice showed granulomas and eosinophilic crystals, with variable degrees of severity and penetrance, that were absent from the lungs from age‐matched wild‐type mice (Table 1; Figure 3A–D). The grading of the lung granulomas and eosinophilic crystals usually went together (i.e. mild granulomas with mild crystals, moderate with moderate, etc.), which suggests that the crystal generation and granuloma formation form part of the same or closely related inflammatory processes in the lung. Previously, Ym1/2 neutrophilic granule proteins have been shown to form eosinophilic crystals in an *Ncf1*‐deficient CGD mouse model, and this was suggested to be due to excess neutrophilic turnover at inflammatory foci 27. The variability in the presence and severity of the granulomas (and crystals) may relate to the age of the mice, presumably because the inability to produce ROS makes the mouse susceptible, but granuloma formation is triggered by environmental events/exposure such as low‐level lung infection that are variable and less predictable.

Interestingly, despite the mice living in the same SPF conditions, the granulomas progressed at a higher rate in *Cyba* ^*tm1a*^ mice than in *Ncf2* ^*tm1a*^ mice, as the majority had to be humanely killed because of difficulty with breathing, and, at necropsy, there were obvious white patches on the lungs, which were histologically confirmed to be granulomas (of moderate–severe grade). Although the ageing study of *Ncf2* ^*tm1a*^ mice was compromised by the majority of them having to be humanely killed at a young age because of scratching, the few *Ncf2* ^*tm1a*^ mice that were not killed at an early age did live past the average age at which the *Cyba* ^*tm1a*^ mice had to be killed becuase of dyspnoea. It is interesting to note that we did not observe the presence of granulomas in the lungs of the bone marrow chimaeras, suggesting that this influx of macrophages resulting in granuloma formation is mediated by a non‐haematopoietic component, possibly endothelial cells; *Cyba* is expressed by endothelial cells, and ROS can participate in vascular function 21.

Thus, in summary, we show that deficiency for any of the five subunits of the NOX2 complex results in decreased pulmonary metastatic colonisation. Focusing on *Cyba*‐deficient and *Ncf2*‐deficient mice revealed that the metastatic colonisation phenotype is mediated by the immune system, and, in support of this, the lungs of these mice showed elevated levels of activated effector T cells and NK cells. The lungs of these mice also showed granulomas even though they were housed in SPF conditions and in *Cyba*‐deficient mice these granulomas led to decreased survival. Thus, the role of NOX2 is complex, and NOX2 deficiency affects multiple aspects of the pulmonary microenvironment that can have both favourable and unfavourable outcomes, which could help to explain the different lung manifestations seen in CGD.

## Author contributions statement

LvdW and AOS conceived the experiments, performed the experiments and wrote the manuscript. A Sw carried out experimental work. SC provided the experimental cohorts of *Cybb* mice and performed the intravenous injections of the bone marrow chimaeras. A Sc and GS provided the experimental cohorts of *Ncf1* mice. MJA carried out the histopathological analysis and provided experimental advice. DJA provided experimental advice and supplied the *Cyba* and *Ncf2* mouse lines. All authors helped to revise the manuscript.

**SUPPLEMENTARY MATERIAL ONLINE**

**Figure S1.** S100+ B16‐F10 melanoma cells are not found within the pulmonary granulomas found in *Cyba*‐deficient mice

## Supporting information


**Figure S1.** S100+ B16‐F10 melanoma cells are not found within the pulmonary granulomas found in *Cyba*‐deficient mice

## References

[1] Gupta GP , Massagué J . Cancer metastasis: building a framework. Cell 2006; 127: 679–695. 17110329 10.1016/j.cell.2006.11.001

[2] Chambers AF , Naumov GN , Varghese HJ , *et al* Critical steps in hematogenous metastasis. an overview Surg Oncol Clin North Am 2001; 10: 243–255. 11382585

[3] Speak AO , Swiatkowska A , Karp NA , *et al* A high‐throughput in vivo screening method in the mouse for identifying regulators of metastatic colonization. Nat Protoc 2017; 12: 2465–2477.2909544210.1038/nprot.2017.118

[4] van der Weyden L , Arends MJ , Campbell AD , *et al* Genome‐wide in vivo screen identifies novel host regulators of metastatic colonization. Nature 2017; 541: 233–236. 28052056 10.1038/nature20792 PMC5603286

[5] van der Weyden L , Karp NA , Swiatkowska A , *et al* Genome wide in vivo mouse screen data from studies to assess host regulation of metastatic colonisation. Sci Data 2017; 4: 170129. 10.1038/sdata.2017.129 PMC5827107 28895944

[6] Brandes RP , Weissmann N , Schröder K . Nox family NADPH oxidases: molecular mechanisms of activation. Free Radic Biol Med 2014; 76: 208–226. 25157786 10.1016/j.freeradbiomed.2014.07.046

[7] Vignais PV . The superoxide‐generating NADPH oxidase: structural aspects and activation mechanism. Cell Mol Life Sci 2002; 59: 1428–1459. 12440767 10.1007/s00018-002-8520-9 PMC11337443

[8] Heyworth PG , Cross AR , Curnutte JT . Chronic granulomatous disease. Curr Opin Immunol 2003; 15: 578–584. 14499268 10.1016/s0952-7915(03)00109-2

[9] O'Neill S , Brault J , Stasia MJ , *et al* Genetic disorders coupled to ROS deficiency. Redox Biol 2015; 6: 135–156. 26210446 10.1016/j.redox.2015.07.009 PMC4550764

[10] Arnold DE , Heimall JR . A review of chronic granulomatous disease. Adv Ther 2017; 34: 2543–2557. 29168144 10.1007/s12325-017-0636-2 PMC5709447

[11] Mahdaviani SA , Mohajerani SA , Rezaei N , *et al* Pulmonary manifestations of chronic granulomatous disease. Expert Rev Clin Immunol 2013; 9: 153–160. 23390946 10.1586/eci.12.98

[12] Nagaraj S , Gupta K , Pisarev V , *et al* Altered recognition of antigen is a mechanism of CD8^+^ T cell tolerance in cancer. Nat Med 2007; 13: 828–835. 17603493 10.1038/nm1609 PMC2135607

[13] Hellstrand K , Asea A , Dahlgren C , *et al* Histaminergic regulation of NK cells. Role of monocyte‐derived reactive oxygen metabolites. J Immunol 1994; 153: 4940–4947. 7963557

[14] Kotsias F , Hoffmann E , Amigorena S , *et al* Reactive oxygen species production in the phagosome: impact on antigen presentation in dendritic cells. Antioxid Redox Signal 2013; 18: 714–729. 22827577 10.1089/ars.2012.4557

[15] Dingjan I , Paardekooper LM , Verboogen DRJ , *et al* VAMP8‐mediated NOX2 recruitment to endosomes is necessary for antigen release. Eur J Cell Biol 2017; 96: 705–714. 28688576 10.1016/j.ejcb.2017.06.007 PMC5641923

[16] Ryder E , Gleeson D , Sethi D , *et al* Molecular characterization of mutant mouse strains generated from the EUCOMM/KOMP‐CSD ES cell resource. Mamm Genome 2013; 24: 286–294. 23912999 10.1007/s00335-013-9467-x PMC3745610

[17] Pollock JD , Williams DA , Gifford MA , *et al* Mouse model of X‐linked chronic granulomatous disease, an inherited defect in phagocyte superoxide production. Nat Genet 1995; 9: 202–209. 7719350 10.1038/ng0295-202

[18] Huang CK , Zhan L , Hannigan MO , *et al* P47(phox)‐deficient NADPH oxidase defect in neutrophils of diabetic mouse strains, C57BL/6J‐m db/db and db/+. J Leukoc Biol 2000; 67: 210–215. 10670582 10.1002/jlb.67.2.210

[19] Ellson CD , Davidson K , Ferguson GJ , *et al* Neutrophils from p40phox−/− mice exhibit severe defects in NADPH oxidase regulation and oxidant‐dependent bacterial killing. J Exp Med 2006; 203: 1927–1937. 16880254 10.1084/jem.20052069 PMC2118373

[20] Johnstone CN , Smith YE , Cao Y , *et al* Functional and molecular characterisation of EO771.LMB tumours, a new C57BL/6‐mouse‐derived model of spontaneously metastatic mammary cancer. Dis Model Mech 2015; 8: 237–251. 25633981 10.1242/dmm.017830 PMC4348562

[21] Zalba G , San José G , Moreno MU , *et al* NADPH oxidase‐mediated oxidative stress: genetic studies of the p22(phox) gene in hypertension. Antioxid Redox Signal 2005; 7: 1327–1336. 16115038 10.1089/ars.2005.7.1327

[22] Okada F , Kobayashi M , Tanaka H , *et al* The role of nicotinamide adenine dinucleotide phosphate oxidase‐derived reactive oxygen species in the acquisition of metastatic ability of tumor cells. Am J Pathol 2006; 169: 294–302. 16816381 10.2353/ajpath.2006.060073 PMC1698756

[23] Aydin E , Johansson J , Nazir FH , *et al* Role of NOX2‐derived reactive oxygen species in NK cell‐mediated control of murine melanoma metastasis. Cancer Immunol Res 2017; 5: 804–811. 28760732 10.1158/2326-6066.CIR-16-0382

[24] Hashimoto A , Pincelli C , Fujioka A , *et al* Relationship between NK cells and granulomatous inflammation in mice. J Clin Lab Immunol 1990; 33: 41–47. 1966942

[25] Nakano Y , Longo‐Guess CM , Bergstrom DE , *et al* Mutation of the Cyba gene encoding p22phox causes vestibular and immune defects in mice. J Clin Invest 2008; 118: 1176–1185. 18292807 10.1172/JCI33835 PMC2248803

[26] Thomas DC , Clare S , Sowerby JM , *et al* Eros is a novel transmembrane protein that controls the phagocyte respiratory burst and is essential for innate immunity. J Exp Med 2017; 214: 1111–1128. 28351984 10.1084/jem.20161382 PMC5379978

[27] Harbord M , Novelli M , Canas B , *et al* Ym1 is a neutrophil granule protein that crystallizes in p47phox‐deficient mice. J Biol Chem 2002; 277: 5468–5475. 11733538 10.1074/jbc.M110635200

